# Cystatin B is a progression marker of human epithelial ovarian tumors mediated by the TGF-β signaling pathway

**DOI:** 10.3892/ijo.2014.2261

**Published:** 2014-01-21

**Authors:** XINGXING WANG, LU GUI, YOUYUAN ZHANG, JIHONG ZHANG, JIMIN SHI, GUOXIONG XU

**Affiliations:** 1 Center Laboratory, Jinshan Hospital, Fudan University, Shanghai 201508;; 2 Department of Pathology, Jinshan Hospital, Fudan University, Shanghai 201508;; 3 Department of Oncology, Shanghai Medical College, Fudan University, Shanghai 200032, P.R. China

**Keywords:** cystatin B, epithelial ovarian cancer, tumorigenesis, transforming growth factor-β signaling, biomarker

## Abstract

Advanced ovarian cancer is a devastating disease. Gaining biomarkers of early detection during ovarian tumorigenesis may lead to earlier diagnosis and better therapeutic strategies. Cystatin B (CSTB) functions as an inhibitor to suppress intracellular cysteine proteases and has been implicated in several types of cancers. The present study explored the expression of CSTB in human ovarian tumors, to investigate CSTB expression associated with clinicopathological features, and to examine the effect of transforming growth factor-β (TGF-β), which plays a key role in ovarian tumorigenesis, on CSTB expression in ovarian cancer cells. The ovarian tissue samples from 33 patients were retrieved. The expression of CSTB in ovarian tissue was examined by immunohistochemistry. We found that CSTB was over-expressed in human ovarian surface epithelial tumors, including serous, mucinous and clear cell tumors. The immunoreactive staining of CSTB was strong in borderline and malignant tumors, weak in benign tumors, and negative in normal tissue counterparts, but was not correlated with the clinicopathological features of patients with ovarian tumors, such as age, histological types, tumor size, lymph node metastasis and clinical stages. The CSTB at mRNA and protein levels in two types of epithelial ovarian cancer cells, OVCAR-3 and SK-OV-3, was decreased after TGF-β1 treatment detected by quantitative PCR and western blot analysis, respectively. The inhibitory effect of TGF-β1 on CSTB expression was abolished in the presence of SB-431542, a TGF-β type I receptor kinase inhibitor. Our data suggest that CSTB is tumor tissue-specific and overexpressed in ovarian borderline and malignant tumors. The increased CSTB expression in ovarian tissue represents tumor progression and is dysregulated by the TGF-β signaling pathway. CSTB may become a novel diagnostic intracellular biomarker for the early detection of ovarian cancer.

## Introduction

Ovarian cancer is the most lethal gynecologic malignancy and the fifth leading cause of cancer death in women (1–3). The estimated newly diagnosed cases of ovarian cancer are 204,000 with 125,000 deaths annually worldwide (1,4). In human, there are three major types of ovarian cancer: epithelia, stromal and germ cell (5). Among these, epithelial ovarian cancer (EOC) accounts for about 85–90% of total ovarian cancers and occurs most commonly in postmenopausal women (2,6). The 5-year survival of patients diagnosed with ovarian cancer at an early stage is significantly different from that of patients with ovarian cancer at an advanced stage. Studies over the past 3 decades show that the 5-year disease-free survival and overall survival of patients with the early stage (stage I) ovarian cancer are 91 and 94%, respectively, with surgery alone (7). However, the 5-year survival of patients with advanced stage disease is <30% (8) and has not enhanced significantly, despite the improvement of various treatments. EOC is highly lethal because it tends to be at an advanced stage upon diagnosis. The low survival rate for women with EOC results in part from an inability to detect the disease at an early curable stage, the lack of effective treatment for the advanced cancer, and our incomplete understanding of how the EOC develops. It is, therefore, essential to identify potential biomarkers and their regulation by the signaling pathways which are possibly altered in the early stages of ovarian cancer. To this effect, we have applied a proteomics study to identify potential biomarkers by comparing BRCA1-mutated immortalized ovarian surface epithelial (OSE) cells, genetically predisposed to ovarian cancer due to the expression of a mutated BRCA1, to wild-type immortalized OSE cells, since women with a germ-line mutation in the BRCA1 gene have a cumulative lifetime risk of 40–50% for ovarian cancer (9). We observed a set of differentially expressed proteins (unpublished data). Among these proteins, cystatin B (CSTB) was found to be increased in BRCA1-mutated cells.

CSTB, also known as stefin B, belongs to the cystatins superfamily which has primarily been explored with respect to its capacity to inhibit intracellular cysteine proteases leaking from lysosomes (10) and has been implicated in several types of cancers, such as breast, lung and colorectal cancers, glioblastoma, squamous cell carcinoma of the head and neck, laryngeal, esophageal and hepatocellular carcinomas, and prostatic adenocarcinoma (11–20). The dysregulated expression of CSTB appears to be associated with tumorigenesis and may be mediated by a variety of cytokines and growth factors, including transforming growth factor-β (TGF-β).

TGF-β has been implicated in many developmental, physiologic and pathologic processes (21), and plays a key role in tumorigenesis of many tissues, including the ovary (22). TGF-β signals through type I and type II serine/threonine kinase receptors and intracellular signaling Smad proteins (23,24). Following receptor activation upon TGF-β stimulation, receptor-regulated Smads, such as Smad2 and Smad3, are phosphorylated and activated, and subsequently form complexes with Smad4 that translocate into the nucleus where they interact with other transcription factors to regulate the transcription of target genes (25), thereby activating downstream signaling cascades. However, accumulated evidence shows that TGF-β signaling is impaired in ovarian cancer. TGF-β1, its receptors (TβR-I and TβR-II), and its signaling proteins (Smad2 and Smad4) have been found to be mutated in ovarian cancer (26,27). Loss-of-function mutations can lead to the disruption of TGF-β-signaling pathways and subsequent loss of cell cycle control (28).

The current study was undertaken to explore the expression of CSTB in human ovarian benign, borderline, and malignant tumors and investigate whether CSTB expression is associated with clinicopathological features of human EOC. Furthermore, we examined for the first time the regulation of CSTB expression mediated by the TGF-β signaling pathway in ovarian cancer cells.

## Materials and methods

### Patients and ovarian tissue samples

The study on human subjects was approved by the Ethics Committee of Jinshan Hospital, Fudan University. The ovarian tissue samples from 27 patients with ovarian tumor and 6 patients with non-ovarian tumor at Jinshan Hospital, Fudan University from January, 2005 to December, 2012 were retrieved for the present study. All patients underwent cytoreductive surgery. None of the patients had received chemotherapy or radiotherapy before surgery. The 10% formalin-fixed paraffin-embedded ovarian tissue specimens were collected and pathologically diagnosed by the Department of Pathology, Jinshan Hospital. The histological examination and classification were based on the current criteria of tumor, node, metastasis (TNM) classification method from the American Joint Committee on Cancer (AJCC) and from the World Health Organization (WHO). Final diagnosis with tumor stage and grade was performed by experienced gynaecologists and pathologists according to the FIGO (International Federation of Gynaecological Oncologists) system. Among the patients, thirteen patients aged 39–66, four patients aged 37 to 50, and ten patients aged 39 to 70 were diagnosed with malignant, borderline and benign tumors, respectively. The normal ovaries from six patients aged 41 to 67 who were diagnosed with non-ovarian diseases were removed and used as controls.

### Immunohistochemical staining and analysis

Four micrometer tissue section was heated at 60°C for 2 h, followed by deparaffinizing in xylene and rehydrating with graded alcohols. For antigen retrieval, the section was immersed in 0.1 M sodium citrate buffer (pH 6.0) and heated in a microwave oven at 100°C for 1.5 min. Endogenous peroxide activity was quenched by 3% hydrogen peroxide in methanol for 15 min. After blocking with 10% normal goat serum (Maixin Bio, Fuzhou, China) for 40 min at room temperature, the section was incubated with a polyclonal rabbit anti-CSTB antibody (1:1,000 dilution, Abcam, Hong Kong, China) at 4°C overnight, followed by incubation with biotinylated anti-rabbit secondary antibody (Maixin Bio) at room temperature for 1 h. After washing, the signal was detected using a DAB Kit (Maixin Bio). Finally, the section was counterstained with hematoxylin and photographed under a light microscope (Nikon, Tokyo, Japan). A normal ovarian tissue without a primary antibody was used as a negative control.

Scoring of CSTB immunoreactive staining was performed by two independent pathologists without any prior knowledge of patient’s clinical data. The proportion of positive cells was scored by the extent of immunoreactive staining to one of the following categories as described previously (29): 0, 0% no positive cells; 1, ≤25% positive cells; 2, 26–50% positive cells; 3, 51–75% positive cells; and 4, >75% positive cells. The intensity of immunoreactive staining was assigned as 0, no staining; 1, weak staining; 2, moderate staining; and 3, strong staining. A final immunoreactive score, also known as the staining index (SI), was determined by the sum of the positive extent and staining intensity. SI was clustered into four groups: ‘0’, ≤2 sum points; ‘1’, 3–4 sum points; ‘2’, 5–6 sum points; and ‘3’, 7 sum points. Finally, for this study, we defined the cases with grades equal to 0 and 1 as CSTB negativity and those with grades equal to 2 and 3 as CSTB positivity.

### Cell culture and TGF-β1 treatment

Human serous ovarian cancer cell lines were purchased from American Type Culture Collection (ATCC, Manassas, VA, USA). OVCAR-3 cells were cultured in RPMI-1640 medium (HyClone, Thermo Fisher Scientific Inc., Beijing, China) supplemented with 20% fetal bovine serum (FBS, Invitrogen, Carlsbad, CA, USA). SK-OV-3 cells were cultured in McCoy’s 5A medium (Sigma, Saint Louis, MO, USA) supplemented with 10% FBS. After seeding for 24 h, the cells were treated with recombinant human TGF-β1 (R&D Systems, Minneapolis, MN, USA) at different doses (0, 0.1, 1 and 10 ng/ml) for 24 h. For a time-course study, OVCAR-3 and SK-OV-3 cells were treated with 10 and 1 ng/ml of TGF-β1, respectively, for 1, 6 and 24 h. A TGF-β type I receptor kinase inhibitor, SB-431542 (30), was obtained from Sigma and dissolved at a concentration of 10 mM in dimethyl sulfoxide (DMSO). For blocking the TGF-β signaling pathway, cells were pre-treated with 10 *μ*M SB-431542 for 30 min and then treated with TGF-β1 for 24 h. DMSO was used as a vehicle control.

### RNA extraction and quantitative real-time PCR

Total RNA in the cells was extracted using TRIzol (Invitrogen), according to the manufacturer’s instruction. One microgram of total RNA was reversely transcribed using a reverse transcription kit (Takara Biotechnology Co., Ltd., Dalian, China). The primer sequences were 5′-CTGTGTTTAAGGCCGTGTCA-3′ (forward) and 5′-AGGTCAGCTCATCATGCTTG-3′ (reverse) for human CSTB and 5′-ACAATGTGGCCGAGGACTTT-3′ (forward) and 5′-GCACGAAGGCTCATCATTCA-3′ (reverse) for human β-actin (synthesized by Sangon Biotech Co., Ltd., Shanghai, China). PCR amplification was performed at 95°C, 5 sec and 60°C, 31 sec for 40 cycles using Takara SYBR Premix Taq^™^ II (Tli RNaseH Plus) kit (Takara Biotechnology Co., Ltd.) with an initial step of denaturing RNA at 95°C for 30 sec. Assays were conducted in triplicate and repeated three times. The amount of target (CSTB) normalized to an endogenous control (β-actin) given by 2^ΔΔCt^, in which threshold cycle (Ct) was obtained using Sequence Detection Software v1.4 (7300 Real Time PCR System, Applied Biosystems, Foster City, CA, USA).

### Western blot analysis

OVCAR-3 and SK-OV-3 cells were lysed in SDS lysis buffer (Beyotime, Haimen, China) with 1% PMSF (Beyotime), followed by sonication. Equal amount of protein was separated on 15% SDS-PAGE and transferred to a PVDF membrane (Millipore, Billerica, MA, USA). After blocking with 5% non-fat milk in Tris-buffered saline with Tween-20 (TBS-T) for 1 h, the membrane was incubated with a primary antibody at 4°C overnight and subsequently incubated with horseradish peroxidase-conjugated goat anti-rabbit or anti-mouse IgG (1:3,000 dilution, Cell Signaling Technology, Inc., Danvers, MA, USA) for 1 h at room temperature. The following primary antibodies were used: rabbit anti-CSTB (1:10,000 dilution, Abcam), mouse anti-Smad 2 (1:2,000 dilution), rabbit anti-phospho-Smad2 (1:2,000 dilution), and rabbit anti-β-actin (1:5,000 dilution) (Cell Signaling Technology, Inc.). Signals were detected using Immobilon™ Western Chemiluminescent HRP Substrate (Millipore) and quantified using Tanon-4500 Gel Imaging System with GIS ID Analysis Software v4.1.5 (Tanon Science & Technology Co., Ltd., Shanghai, China).

### Statistical analyses

All analyses were performed with SPSS Statistics 19.0 for Windows (SPSS, Chicago, IL, USA). For comparison between two groups of positivity and correlation between CSTB expression and histological types or the clinicopathological characteristics, a Fisher’s exact test was performed. For comparison between the two scoring groups of immunostaining, a Wilcoxon rank-sum test was applied. For comparison between two groups in the treatment experiments, a Student’s t-test was used. Results are presented as the mean ± standard error of mean (SEM). Significant difference was considered at the value of P<0.05.

## Results

### Overexpression of CSTB in human ovarian tumors

**Figure 1.**
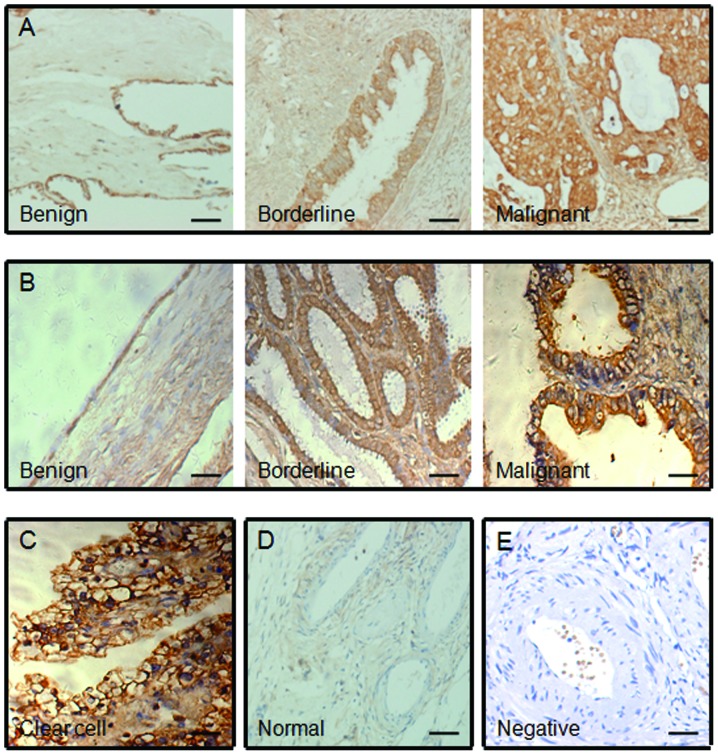
Immunohistochemical staining of CSTB protein in human ovarian tissues. Representative images of CSTB expression in (A) ovarian serous, (B) mucinous and (C) clear cell tumors, and (D) the normal ovarian tissue are shown. Benign, benign tumor; borderline, borderline tumor; malignant, malignant tumor; clear cell, clear cell malignant tumor; normal, normal ovarian tissue; negative, negative control without first antibody in the (E) normal ovarian tissue. A brown color in epithelial cell is considered as a positive staining. Original magnification, ×200. Scale bar, 100 *μ*m.

By immunohistochemistry staining, the overexpression of CSTB was observed in human ovarian tumors, including benign, borderline and malignant tumors, albeit at different levels (Fig. 1A, B and C), compared with the normal ovarian tissues that showed negative staining (Fig. 1D). The positive staining (brown color) was mainly distributed in the cytoplasm of the epithelial cells of ovarian tumor and the expression of CSTB protein was weak in benign tumors, moderate in borderline tumors, and strong in malignant tumors. Indeed the highest intensity of staining was found in ovarian serous, mucinous and clear cell malignant tumors (Fig. 1A, B and C). Based on the SI system as indicated before, we classified CSTB expression into positive and negative categories (Table I). By comparison of ovarian tumors with the normal ovarian tissue, we found that the positive rate of CSTB expression was different. The positive rate of CSTB expression was significantly higher in tumors, including benign, borderline and malignant tumors, than that in normal tissue (P<0.01), though there was no difference in the CSTB-positive rate between benign, borderline and malignant tumors (P>0.05) (Table I). However, statistical analysis of the immunoreactive scores of CSTB protein between the types of tissues indicated that the expression of CSTB protein was different between the four groups: the normal ovarian tissue, ovarian benign tumor, ovarian borderline tumor and ovarian malignant tumor. The immunoreactive score of CSTB protein is significantly higher in the tumors than that in the normal tissue (all P<0.01) (Table II). Furthermore, by comparison of benign, borderline and malignant tumors, we observed that the immunoreactive score of CSTB protein was significantly higher in borderline and malignant tumors than that in benign tumors (both P<0.05). The expression of CSTB protein tended to be high in malignant tumors, but there was no significant difference of immunoreactive score of CSTB protein between borderline and malignant tumors (P>0.05). These data suggest that CSTB is an ovarian tumor marker and an increase in the expression of CSTB in ovarian tissue represents tumor progression.

### Correlation of CSTB expression with clinicopathological features

Next we examined whether the expression of CSTB is correlated with the clinicopathological features of patients with epithelial ovarian tumors. The histopathological characteristics are listed in Table III. All information was gathered by reviewing medical charts and pathology records. By comparison of CSTB protein expression associated with age, we found that CSTB expression was not significantly different between younger (≤45 years) and older (>45 years) patients with ovarian tumor (P>0.05). The positive rate of CSTB expression was 100% in mucinous and clear cell tumors and 66.67% in serous tumor, including benign, borderline and malignant tumors (Table III). However, multiple comparisons of the histological types showed that there was no difference of CSTB expression between serous, mucinous and clear cell tumors (P>0.05). By comparison of CSTB expression associated with tumor size, the positivity of CSTB expression was not significantly different between small size (≤2 cm) and large size (>2 cm) of tumors (total and malignant, both P>0.05). We examined the involvement of lymph nodes on CSTB expression and found that the CSTB protein was not associated with lymph node metastasis (P>0.05). Multiple comparison of clinical stages revealed that there was no difference in the positivity of CSTB expression. Because the sample number of malignant tumors was relatively small (total 13 cases), an increase in sample number must be considered in future study. Overall, these data indicate that CSTB is a tumor marker and there is no correlation of CSTB expression with clinicopathological features of ovarian cancer patients, such as age, histological types, tumor size, lymph node metastasis and clinical stages.

### Regulation of CSTB expression by TGF-β1 in ovarian cancer cells

**Figure 2.**
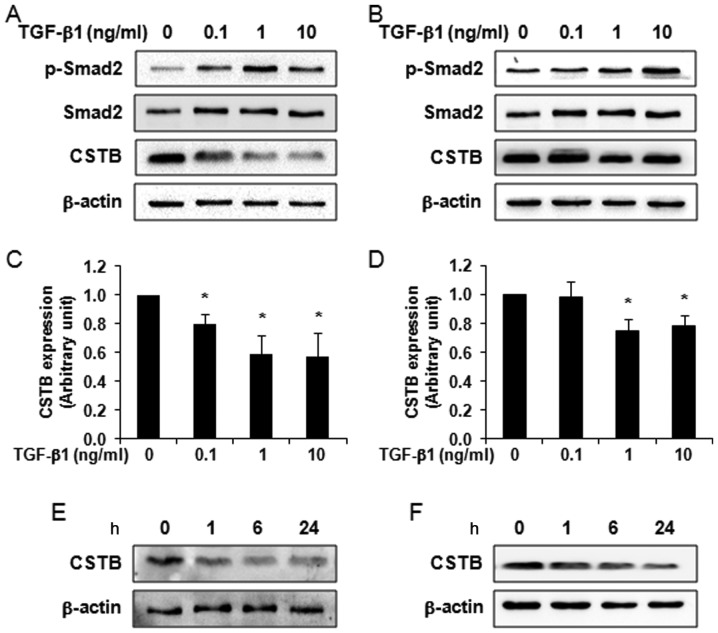
Effect of TGF-β1 on the expression of CSTB protein in ovarian cancer cells. (A) OVCAR-3 and (B) SK-OV-3 cells were treated with TGF-β1 at different concentrations (0.1, 1, 10 ng/ml) for 24 h. Equal amounts of total protein were subjected to SDS-PAGE and transferred to a PVDF membrane. Specific signal was detected by western blot analysis using a specific antibody against phospho-Smad2, total Smad2, CSTB or β-actin. Histograms show the quantitative analyses of the gels from (C) OVCAR-3 (n=4) and (D) SK-OV-3 (n=5) cells after densitometry. CSTB expression was decreased by TGF-β1 treatment in a dose-dependent manner (^*^P<0.05). In a time-course study, (E) OVCAR-3 and (F) SK-OV-3 cells were treated with 10 and 1 ng/ml of TGF-β1, respectively, for 1, 6 and 24 h (both n=2).

Since TGF-β plays a key role in ovarian tumorigenesis and since CSTB was overexpressed in ovarian cancer, we subsequently investigated whether TGF-β1 affects CSTB protein expression. The dose-dependent and time-course studies of TGF-β1 in two epithelial ovarian cancer cell lines, OVCAR-3 and SK-OV-3, were applied. Because Smad2 is a TGF-β signaling protein and activated upon TGF-β1 treatment (25), first of all we detected the phosphorylation of Smad2 by western blot analysis to confirm the responsiveness of tested cells to TGF-β1. Indeed we found that TGF-β1 at different doses (ranged 0.1–10 ng/ml) increased the phosphorylation of Smad2 in OVCAR-3 (Fig. 2A) and SK-OV-3 (Fig. 2B) cells. After stripping, we reprobed the same blot with anti-CSTB antibody and found that TGF-β1 significantly decreased CSTB in OVCAR-3 (Fig. 2A and C) and SK-OV-3 (Fig. 2B and D) cells in a dose-dependent manner. The time-course study also showed that CSTB expression was decreased in OVCAR-3 (Fig. 2E) and SK-OV-3 (Fig. 2F) cells after 10 and 1 ng/ml of TGF-β1 treatment, respectively.

**Figure 3.**
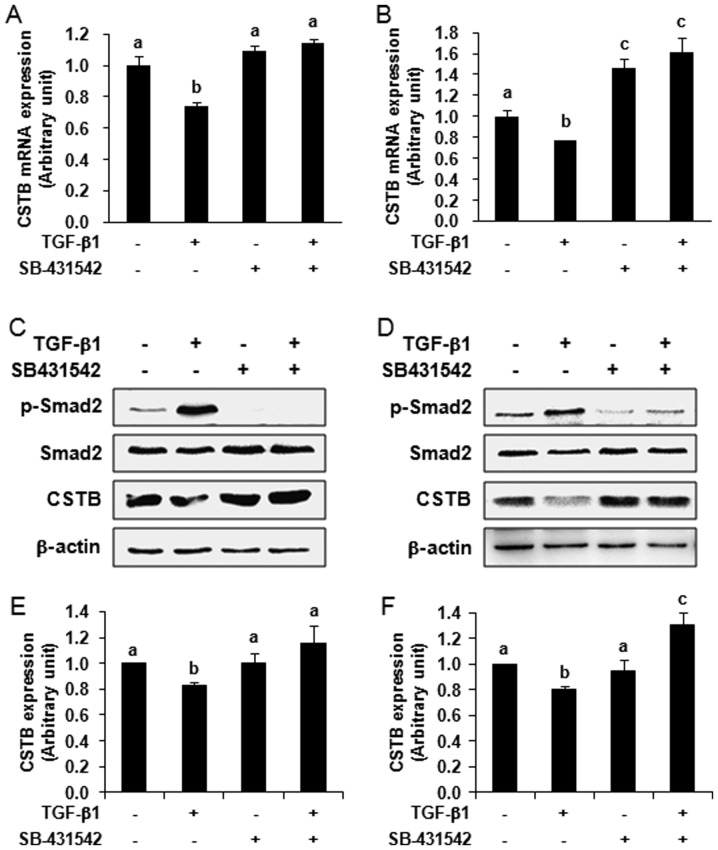
Regulation of the expression of CSTB by the TGF-β signaling pathway in ovarian cancer cells. (A, C and E) OVCAR-3 and (B, D and F) SK-OV-3 cells were pre-treated with a TGF-β type I receptor kinase inhibitor (10 *μ*M SB431542) for 30 min and then treated with 10 and 1 ng/ml of TGF-β1, respectively, for 24 h. CSTB mRNA was detected by quantitative real-time PCR using primers specific to CSTB (A and B; both n=3). CSTB protein was detected by western blot analysis using a specific antibody against phospho-Smad2, total Smad2, CSTB or β-actin (C and D). Histograms show the quantitative analyses of the gels from (E) OVCAR-3 (n=4) and (F) SK-OV-4 (n=3) cells after densitometry. Data with different superscripts were significantly different from each other (P<0.05), whereas those with the same superscripts were not.

Next, we blocked the TGF-β signaling pathway by treating ovarian cancer cells with a special TGF-β type I receptor kinase inhibitor, SB-431542, to see if the regulation of CSTB expression is mediated by the TGF-β signaling pathway. By comparing with the vehicle control group, we found that TGF-β1 had an inhibitory effect on the expression of CSTB mRNA. The expression of CSTB mRNA was significantly decreased after TGF-β1 treatment in OVCAR-3 (Fig. 3A) and SK-OV-3 (Fig. 3B) cells detected by qPCR (P<0.05). This downregulation of CSTB mRNA expression by TGF-β1 was abolished after pre-treatment of cells with 10 *μ*M SB-431542 for 30 min. In SK-OV-3 cells, we observed an increase of CSTB expression after SB-431542 pre-treatment, which may be due to the elimination of the inhibitory effect of endogenous TGF-β on CSTB expression. At the protein level, we observed the phosphorylation of Smad2 in OVCAR-3 (Fig. 3C) and SK-OV-3 (Fig. 3D) cells upon 10 and 1 ng/ml of TGF-β1 treatment, respectively, for 24 h. This phosphorylation was abolished in the cells pre-treated with 10 *μ*M SB-431542 for 30 min, indicating the responsiveness of the cells to this inhibitor. After stripping, the same blots were reprobed with anti-CSTB antibody. We found that the expression of CSTB protein was significantly decreased after TGF-β1 treatment (P<0.05) and the decrease of CSTB by TGF-β1 was blocked by the pre-treatment of SB-431542 in OVCAR-3 (Fig. 3C and E) and SK-OV-3 (Fig. 3D and F) cells. Thus, our data indicate that the regulation of CSTB expression is mediated by the TGF-β signaling pathway.

## Discussion

In the present study we revealed the expression of CSTB associated with clinicopathological features in human epithelial ovarian cancer and examined for the first time the expression of CSTB regulated by the TGF-β signaling pathway in ovarian cancer cells. CSTB was initially found in ascites fluid from patients with ovarian carcinoma by Lah *et al* in 1992 (31) and so far this is the only group to show the expression of CSTB in ovarian cancer. Here we demonstrated that CSTB protein was indeed not only overexpressed in epithelial ovarian malignant tumor, but also expressed in benign and borderline tumors; the latter was not reported previously.

Serous carcinoma, arising from the ovarian surface epithelium (OSE) and/or fallopian tube epithelium (FTE), is the most frequent ovarian cancer. Although the detection of CSTB in ovarian serous malignant tumor has been reported (31,32), this is the first report showing that CSTB was also expressed in mucinous and clear cell tumors. Furthermore, we observed the overexpression of CSTB in benign and borderline tumors, comparing with normal tissue counterparts which appeared negative, suggesting that CSTB is tumor tissue-specific. However, the function and the role of CSTB in ovarian tumorigenesis remain unclear. CSTB is one of the endogenous inhibitors of lysosomal cysteine proteases and thought to play a role in protecting against the proteases leaking from lysosomes. Alterations in CTSB expression have been found at various diseases, including epilepsy and cancer. CSTB mutations are responsible for progressive myoclonus epilepsy type 1 (EPM1) (33). CSTB-null mice can develop symptoms that mimic EPM1 (34). In cancer research, CSTB deficiency reduces tumor growth via the sensitization of tumor cells to oxidative stress in a breast cancer model (35). CSTB deficiency in these mice results in enhanced cathepsin B and D activities, indicating lysosomal dysfunction. On the other hand, increased CSTB has been observed in various cancers such as lung, hepatocellular and colorectal cancers (17–19). It has been reported that CSTB, derived from serous ovarian carcinomas, strongly inhibits papain and cathepsin L and moderately inhibits cathepsin B (32). These results imply an *in vivo* role for CSTB in tumorigenesis. An imbalance between intracellular cathepsins and CSTB may facilitate the progression of ovarian epithelial cell transformation.

By comparing the clinicopathological features of patients with epithelial-type tumors of the ovary, we found that CSTB was not correlated with age, histological types, tumor size and stage, and lymph node metastasis. Although the number of cases in this study was relatively small (total 27 patients with ovarian tumor), our data were similar to the results obtained from a lung cancer study that the high concentration of CSTB in human lung tumor tissue specimen is not correlated with TNM stages, but positively correlated with survival probability (17). However, in bladder cancer, urine levels of CSTB are positively correlated with tumor grade, stage and shorter time to disease recurrence and progression (36). During the preparation of this manuscript, a group from Russia reported the elevation of serum and ascites CSTB in ovarian cancer patients (37). Overall, these studies indicate that CSTB may be useful as an ovarian tumor marker and a target protein for diagnosis, prognosis and therapy in cancer. Therefore, the follow-up of patients with an ovarian tumor and the measurement of the serum and urine levels of CSTB in patients may be of great interest and should be proposed as the next investigation.

Although the overexpression of CSTB in various cancers is observed, the mechanisms underlying the regulation of CSTB in cancer progression are unknown. Because the growth inhibitory effect of TGF-β prevents overproliferation of OSE during wound healing after ovulation, the dysregulation of TGF-β signaling is thought to be crucial to the development of EOC (28,38). Ovarian cancer at early stage is refractory to TGF-β-mediated growth inhibition, whereas at later stage TGF-β promotes tumor proliferation and epithelial-mesenchymal transition (EMT) (22,38–40). However, whether the expression of CSTB in ovarian tumor is regulated by the TGF-β signaling pathway remains unclear. Our *in vitro* study showed that CSTB expression in two epithelial ovarian cancer cell lines was decreased after TGF-β1 treatment. By blocking the TGF-β signaling pathway using an inhibitor to TGF-β type I receptor, we demonstrated for the first time that the inhibition of TGF-β signaling resulted in the abolishment of CSTB suppression, suggesting that the regulation of CSTB is mediated by the TGF-β signaling pathway. It has been reported that alteration of TGF-β components is crucial for ovarian tumorigenesis (26–28,41,42). We speculate that in *in vivo* circumstance the loss of TGF-β responsiveness or the defectiveness of TGF-β signaling may lead to the upregulation of CSTB, while the TGF-β-mediated CSTB-inhibitory pathway can be interrupted in the ligand-receptor-Smad axis, such as insufficient secretion and activation of TGF-β1, mutational inactivation of its receptors or Smad proteins, and misconducted signal transduction.

In conclusion, CSTB was overexpressed in human epithelial ovarian tumors, including serous, mucinous and clear cell tumors. The immunoreactive staining of CSTB was negative in normal tissue, weak in benign tumor and strong in borderline and malignant tumors, which may represent tumor progression. CSTB at mRNA and protein levels was regulated by the TGF-β signaling pathway. Our data suggest that CSTB is tumor tissue-specific and a potential diagnostic marker for ovarian cancer. Gaining better understanding of how TGF-β regulates the CSTB expression during ovarian tumorigenesis may lead to better therapeutic strategies by targeting CSTB for this devastating disease. The follow-up of patients and the examination of the serum and urine CSTB in a larger population of patients with ovarian tumor may be of great interest in subsequent studies.

## Figures and Tables

**Table I.** CSTB protein expression in human ovarian tissues.

	n	CSTB expression		CSTB positive rate (%)
		Positive	Negative	
Normal	6	0	6	0.00
Benign	10	8	2	80.00
Borderline	4	4	0	100.00
Malignant	13	12	1	92.31

Based on the SI system, the positivity and negativity categories were classified. For comparison between 2 groups, a Fisher’s exact test was applied. n, number of cases; normal, normal ovarian tissue; benign, ovarian benign tumor; borderline, ovarian borderline tumor; malignant, ovarian malignant tumor. Statistical analysis: normal vs. benign, P=0.003; normal vs. borderline, P=0.005; normal vs. malignant, P<0.001; benign vs. borderline, P=0.495; benign vs. malignant, P=0.398; borderline vs. malignant, P=0.765.

**Table II.** Comparison of CSTB immunostaining in the ovarian tissues.

Comparison	Z score	P-value
Normal vs. benign	−3.422	0.001
Normal vs. borderline	−2.631	0.009
Normal vs. malignant	−3.211	0.001
Benign vs. borderline	−2.286	0.022
Benign vs. malignant	−2.319	0.020
Borderline vs. malignant	0.238	0.812

Wilcoxon rank-sum test was used to analyze CSTB immunoreactive scores between two types of tissues. Normal, normal ovarian tissue; benign, ovarian benign tumor; borderline, ovarian borderline tumor; malignant, ovarian malignant tumor.

**Table III.** Clinicopathological features of patients with epithelial-type ovarian tumors correlated with CSTB expression detected by immunohistochemistry.

Clinico-pathological features	n	CSTB expression		P-value
		Positive (%)	Negative (%)	
Age at diagnosis				0.669
≤45	8	7 (87.50)	1 (12.50)	
>45	19	17 (89.47)	2 (10.53)	
Histological type				0.492^a^
Serous tumor				
Benign	6	4 (66.67)	2 (33.33)	
Borderline	2	2 (100.00)	0 (0.00)	
Malignant	7	6 (85.71)	1 (14.29)	
Mucinous tumor				
Benign	4	4 (100.00)	0 (0.00)	
Borderline	2	2 (100.00)	0 (0.00)	
Malignant	3	3 (100.00)	0 (0.00)	
Clear cell tumor	3	3 (100.00)	0 (0.00)	
Tumor size				0.308^b^
Benign				
≤2 cm	0	0 (0.00)	0 (0.00)	
>2 cm	10	8 (80.00)	2 (20.00)	
Borderline				
≤2 cm	0	0 (0.00)	0 (0.00)	
>2 cm	4	4 (100.00)	0 (0.00)	
Malignant				0.231
≤2 cm	3	2 (66.67)	1 (33.33)	
>2 cm	10	10 (100.00)	0 (0.00)	
LN metastasis				0.769
Yes	3	3 (100.00)	0 (0.00)	
No	10	9 (90.00)	1 (10.00)	
FIGO stage				0.154^c^
I	5	5 (100.00)	0 (0.00)	
II	1	0 (0.00)	1 (100.00)	
III	6	6 (100.00)	0 (0.00)	
IV	1	1 (100.00)	0 (0.00)	

For comparison of CSTB protein expression associated with age, histological type, tumor size, lymph node metastasis, and FIGO stage, Fisher’s exact test was applied. n, number of cases; LN, lymph node.

a Multiple comparison of the histological types.

b Tumor size comparison (≤2 cm vs. >2 cm, total).

c Multiple comparison of the stages.

## Abbreviations:

CSTB: cystatin B;
TGF-β: transforming growth factor-β;
EOC: epithelial ovarian cancer;
OSE: ovarian surface epithelial;
TNM: tumor, node, metastasis;
IHC: immunohistochemistry;
qPCR: quantitative polymerase chain reaction;
SI: staining index

## References

[1] Rauh-Hain JA, Krivak TC, Del Carmen MG, Olawaiye AB (2011). Ovarian cancer screening and early detection in the general population. Rev Obstet Gynecol.

[2] Auersperg N, Wong AS, Choi KC, Kang SK, Leung PC (2001). Ovarian surface epithelium: biology, endocrinology, and pathology. Endocr Rev.

[3] Siegel R, Naishadham D, Jemal A (2013). Cancer statistics, 2013. CA Cancer J Clin.

[4] Sankaranarayanan R, Ferlay J (2006). Worldwide burden of gynaecological cancer: the size of the problem. Best Pract Res Clin Obstet Gynaecol.

[5] Burger HG, Fuller PJ, Chu S (2001). The inhibins and ovarian cancer. Mol Cell Endocrinol.

[6] Jelovac D, Armstrong DK (2011). Recent progress in the diagnosis and treatment of ovarian cancer. CA Cancer J Clin.

[7] Jelovac D, Armstrong DK (2011). Role of farletuzumab in epithelial ovarian carcinoma. Curr Pharm Des.

[8] Van Nagell JR, Pavlik EJ (2012). Ovarian cancer screening. Clin Obstet Gynecol.

[9] Prat J, Ribe A, Gallardo A (2005). Hereditary ovarian cancer. Hum Pathol.

[10] Turk V, Stoka V, Turk D (2008). Cystatins: biochemical and structural properties, and medical relevance. Front Biosci.

[11] Smid L, Strojan P, Budihna M (1997). Prognostic value of cathepsins B, D and steffins A and B in laryngeal carcinoma. Eur Arch Otorhinolaryngol.

[12] Kos J, Lah TT (1998). Cysteine proteinases and their endogenous inhibitors: target proteins for prognosis, diagnosis and therapy in cancer (Review). Oncol Rep.

[13] Levicar N, Kos J, Blejec A (2002). Comparison of potential biological markers cathepsin B, cathepsin L, stefin A and stefin B with urokinase and plasminogen activator inhibitor-1 and clinicopathological data of breast carcinoma patients. Cancer Detect Prev.

[14] Zhang R, Tremblay TL, McDermid A, Thibault P, Stanimirovic D (2003). Identification of differentially expressed proteins in human glioblastoma cell lines and tumors. Glia.

[15] Strojan P, Anicin A, Svetic B, Pohar M, Smid L, Kos J (2007). Stefin A and stefin B: markers for prognosis in operable squamous cell carcinoma of the head and neck. Int J Radiat Oncol Biol Phys.

[16] Shiraishi T, Mori M, Tanaka S, Sugimachi K, Akiyoshi T (1998). Identification of cystatin B in human esophageal carcinoma, using differential displays in which the gene expression is related to lymph-node metastasis. Int J Cancer.

[17] Ebert E, Werle B, Julke B (1997). Expression of cysteine protease inhibitors stefin A, stefin B, and cystatin C in human lung tumor tissue. Adv Exp Med Biol.

[18] Ji NY, Kang YH, Park MY (2011). Development of a fluorescent microsphere immunoassay for cystatin B (CSTB) in serum of patients with hepatocellular carcinoma. Clin Chem Lab Med.

[19] Kos J, Krasovec M, Cimerman N, Nielsen HJ, Christensen IJ, Brunner N (2000). Cysteine proteinase inhibitors stefin A, stefin B, and cystatin C in sera from patients with colorectal cancer: relation to prognosis. Clin Cancer Res.

[20] Mirtti T, Alanen K, Kallajoki M, Rinne A, Soderstrom KO (2003). Expression of cystatins, high molecular weight cytokeratin, and proliferation markers in prostatic adenocarcinoma and hyperplasia. Prostate.

[21] Massague J (2008). TGFbeta in cancer. Cell.

[22] Nilsson EE, Skinner MK (2002). Role of transforming growth factor beta in ovarian surface epithelium biology and ovarian cancer. Reprod Biomed Online.

[23] Attisano L, Wrana JL (2002). Signal transduction by the TGF-beta superfamily. Science.

[24] Derynck R, Zhang Y, Feng XH (1998). Smads: transcriptional activators of TGF-beta responses. Cell.

[25] Massague J, Seoane J, Wotton D (2005). Smad transcription factors. Genes Dev.

[26] Cardillo MR, Yap E, Castagna G (1997). Molecular genetic analysis of TGF-beta1 in ovarian neoplasia. J Exp Clin Cancer Res.

[27] Wang D, Kanuma T, Mizunuma H (2000). Analysis of specific gene mutations in the transforming growth factor-beta signal transduction pathway in human ovarian cancer. Cancer Res.

[28] Berchuck A, Rodriguez G, Olt G (1992). Regulation of growth of normal ovarian epithelial cells and ovarian cancer cell lines by transforming growth factor-beta. Am J Obstet Gynecol.

[29] Zeng F, Xu G, Zhou T (2012). Reduced expression of activin receptor-like kinase 7 in breast cancer is associated with tumor progression. Med Oncol.

[30] Inman GJ, Nicolas FJ, Callahan JF (2002). SB-431542 is a potent and specific inhibitor of transforming growth factor-beta superfamily type I activin receptor-like kinase (ALK) receptors ALK4, ALK5, and ALK7. Mol Pharmacol.

[31] Lah TT, Kokalj-Kunovar M, Kastelic L (1992). Cystatins and stefins in ascites fluid from ovarian carcinoma. Cancer Lett.

[32] Kastelic L, Turk B, Kopitar-Jerala N (1994). Stefin B, the major low molecular weight inhibitor in ovarian carcinoma. Cancer Lett.

[33] Pennacchio LA, Lehesjoki AE, Stone NE (1996). Mutations in the gene encoding cystatin B in progressive myoclonus epilepsy (EPM1). Science.

[34] Kaur G, Mohan P, Pawlik M (2010). Cystatin C rescues degenerating neurons in a cystatin B-knockout mouse model of progressive myoclonus epilepsy. Am J Pathol.

[35] Butinar M, Prebanda MT, Rajkovic J (2013). Stefin B deficiency reduces tumor growth via sensitization of tumor cells to oxidative stress in a breast cancer model. Oncogene.

[36] Feldman AS, Banyard J, Wu CL, McDougal WS, Zetter BR (2009). Cystatin B as a tissue and urinary biomarker of bladder cancer recurrence and disease progression. Clin Cancer Res.

[37] Gashenko EA, Lebedeva VA, Brak IV, Tsykalenko EA, Vinokurova GV, Korolenko TA (2013). Evaluation of serum procathepsin B, cystatin B and cystatin C as possible biomarkers of ovarian cancer. Int J Circumpolar Health.

[38] Wong AS, Leung PC (2007). Role of endocrine and growth factors on the ovarian surface epithelium. J Obstet Gynaecol Res.

[39] Akhurst RJ, Derynck R (2001). TGF-beta signaling in cancer - a double-edged sword. Trends Cell Biol.

[40] Derynck R, Akhurst RJ, Balmain A (2001). TGF-beta signaling in tumor suppression and cancer progression. Nat Genet.

[41] Lynch MA, Nakashima R, Song H (1998). Mutational analysis of the transforming growth factor beta receptor type II gene in human ovarian carcinoma. Cancer Res.

[42] Chen T, Triplett J, Dehner B (2001). Transforming growth factor-beta receptor type I gene is frequently mutated in ovarian carcinomas. Cancer Res.

